# Performance of Exoelectrogenic Bacteria Used in Microbial Desalination Cell Technology

**DOI:** 10.3390/ijerph17031121

**Published:** 2020-02-10

**Authors:** Li Guang, Desmond Ato Koomson, Huang Jingyu, David Ewusi-Mensah, Nicholas Miwornunyuie

**Affiliations:** 1 School of Environment, Northeast Normal University, Changchun 130024, China; 2 Key Laboratory of Songliao Aquatic Environment, Ministry of Education, Jilin Jianzhu University, Changchun 130118, China; 3 Ministry of Education Key Laboratory of Integrated Regulation and Resource Development on Shallow Lakes, College of Environmental Engineering, Hohai University, No. 1 Xikang Road, Nanjing 210098, China

**Keywords:** microbial desalination cell, exoelectrogens, tri-functional process, mixed culture, predominant species, pure cultures, electron transport chain

## Abstract

The tri-functional purpose of Microbial Desalination Cell (MDC) has shown a great promise in our current scarcity of water, an increase in water pollution and the high cost of electricity production. As a biological system, the baseline force that drives its performance is the presence of exoelectrogens in the anode chamber. Their presence in the anodic chamber of MDC systems enables the treatment of water, desalination of seawater, and the production of electrical energy. This study reviews the characteristics of exoelectrogens, as a driving force in MDC and examines factors which influence their growth and the performance efficiency of MDC systems. It also addresses the efficiency of mixed cultures with certain predominant species as compared to pure cultures used in MDC systems. Furthermore, the study suggests the need to genetically modify certain predominant strains in mixed cultures to enhance their performance in COD removal, desalination and power output and the integration of MDC with other technologies for cost-effective processes.

## 1. Introduction

**Figure 1:**
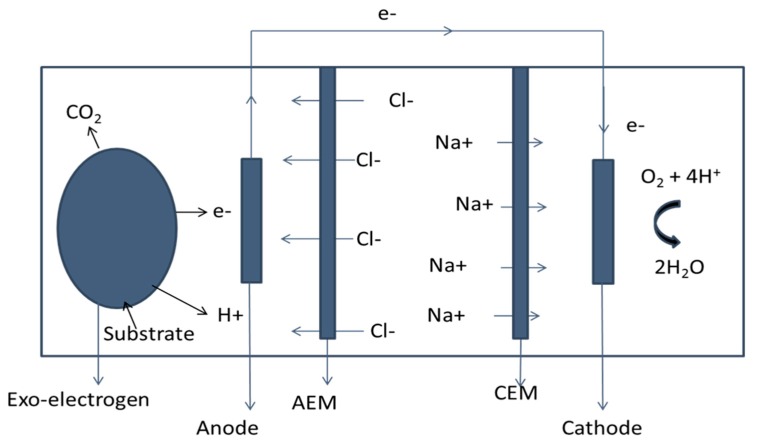
Schematic diagram of a conventional Microbial Desalination Cell (MDC) system with Anode Exchange Membrane (AEM) and Cathode Exchange Membrane (CEM) depicting the general procedure of transfer of electrons from an exo-electrogenic cell after the oxidation of organic matter (substrate) by the cell.

Microbial Desalination Cell (MDC), a tri-functional modern technology, developed for the treatment of wastewater, desalination, and production of electrical energy, was first proposed in 2009 by Cao [1]. A conventional MDC is a three-chamber system with ion-exchange membranes (IEMs), which has a desalination chamber in the middle, as described in Figure 1. In recent years, various types of MDC systems have been designed and introduced [2,3,4,5,6,7,8,9]. Just as Bio-electrochemical systems (BES) have their limitations, the MDC system, upon its inception, was seen to have some limitations such as high internal resistance, pH imbalance, biofouling among others. Numerous studies have extensively investigated and addressed these limitations and more [4,10,11,12]. As a biological technology, a vital part of these systems is the presence of exoelectrogens in the anodic chamber where the performance of the MDC system mainly depends on these exoelectrogens.

Exoelectrogens are microorganisms, mostly bacteria, which generate electrical energy by the oxidation of organic matter and transferring the electrons to an electron acceptor outside of their cells, hence the word “*Exo*”. Which part of the environment serves as a rich source of exoelectrogens? Usually, anaerobic sludge from industrial or domestic wastewater treatment plants, anaerobic sediment, primary industrial or municipal effluent and even farm soil contain the exoelectrogens that can be isolated from the respective sources either as pure culture or mixed culture and can be further used in the MDCs. They can generate electrical energy from organic or chemical sources such as simple carbohydrates, like glucose [13], wastes from municipal, domestic, and industrial wastewater treatment plants have been also used as the carbon source in MDCs [10,13,14] or industrial dyes [15]. Since bacteria are self-replicating, the degradation of organic matter is done without the need of replenishing catalysts. Exoelectrogens are one of the major determiners of the efficiency of MDC systems. Hence, their optimal growth and survival are very important. These bacteria are characterized by many factors, such as salt concentration, temperature, pH, and media [16]. The right exoelectrogens used in an MDC system can enhance its efficiency, especially in the production of electrical energy.

Exoelectrogens that are well known can be categorized into various functional groups based on types of anaerobic respiration [17]. These exoelectrogens include nitrate-reducing bacteria (denitrifying bacteria (DNB)) such as Pseudomonas [18] and Ochrobactrum [19], and dissimilatory metal-reducing bacteria (DMRB), such as Geobacter [20], Shewanella [21], Geopsychrobacter [22], and Geothrix [23], sulfate-reducing bacteria (SRB) including Desulfuromonas [24] and Desulfobulbus [25]. Moreover, through anaerobic respiration pathways, fermentative bacteria, such as Clostridium [26] and *Escherichia coli,* produce electricity [27]. In MFC, purple non-sulfur bacteria, non-photosynthetic *Rhodoferax ferrireducens*, and photosynthetic *Rhodopseudomonas palustris DX-1* have also been found to generate electricity via anaerobic respiration [28,29]. The catabolic and respiratory pathways of exoelectrogens have been suggested to change due to the shifts in electrode potentials [30]. Zhu [31] reported on the need for optimum electrode potential for more efficient production of power by the exoelectrogens. Therefore, the electrode potential stimulating the formation of electro-active biofilm for the production of energy varies widely in the microbial community [32].

Characterization of exoelectrogens used in other BES, such as Microbial Fuel Cells (MFCs), has been well documented [16,31,32,33,34]. Understanding the metabolic activities of exoelectrogens and how their mechanisms influence the overall performance of MDC is very imperative in the scaling and development of the technology [31,32,33]. Over a decade, since the introduction of MDC, intensive studies have been done on its wastewater treatment, desalination, and power generation processes as well as its architecture and mode of operation [1,2,5,6,10,11,12]. Kim and Logan (2013) reviewed state of the art in MDC design and performance and the safety issues related to the use of MDCs with little focus on the types of exoelectrogens and their performances [5]. A similar review study, Huang et al., (2017), looked at the relationship between individual factors and how they contribute to the performance and efficiency of MDCs [4]. Saeed et al., (2015) also made a general review of the MDC technology, the working principle behind the conventional MDC system, and the various types of MDCs that are currently operational [11]. Though MDC technology has received significant research attention over the years, the biological driving-force has not been understood yet. While only a few studies make mention of their predominant species and how such species drive the processes in the cell, most studies discuss just the source of their culture MDC [10,12,13,14,15]. There is a wide loophole in understanding, in detail, the nature, and mechanism of these exoelectrogens used in MDC and their influence on the system, as well as their characterization. This study reviews the characteristics of exoelectrogens, as a driving-force in MDC and examines factors such as pH, desalination, substrate, and power output, which affect their growth and the performance efficiency of MDC systems. It probes the efficiency of mixed cultures with certain predominant species as compared to pure cultures used in MDC systems and outlines significant aspects of further investigation exoelectrogens in MDC and other related fields.

## 2. Characterization of Exoelectrogenic Bacteria in MDC

Pure strains or cultures and mixed cultures of exoelectrogens are generally used in MDC [13,14,15,35]. These cultures, depending on environmental factors behave differently [17,21,36,37,38]. This section discusses their differences in performance, the catabolic and respiratory pathways which involve the Extra-cellular Electron Transfer (EET) mechanisms of these exoelectrogens [32].

### 2.1. Respiration of Exoelectrogens

Generally, bacteria derive energy in the form of Adenosine 5′ Tri-Phosphate (ATP) by two main mechanisms which are substrate-level phosphorylation and oxidative or photo-phosphorylation. The first of these is the formation of adenosine 5′-triphosphate (ATP) by substrate-level phosphorylation and two distinct classes of reaction can be distinguished:

(i) ADP + substrate-P = ATP + substrate(1)

(ii) ADP + Pi + substrate-X = ATP + substrate + X(2)

where ADP is adenosine 5′-diphosphate, and Pi is inorganic phosphate.

In oxidative or photo-phosphorylation, ATP synthesis is coupled to electron transport reactions which, in turn, can be driven by light (in phototrophs) or by the oxidation of both organic compounds (in organo-heterotrophs) and inorganic ions (in chemo-lithotrophs) of negative redox potential, linked to the reduction of electron acceptors of more positive redox potential. Although there are small differences, the overall features of electron transport-dependent ATP synthesis are very similar in bacteria, in mitochondria, and photosynthetic systems [17,30].

**Figure 2:**
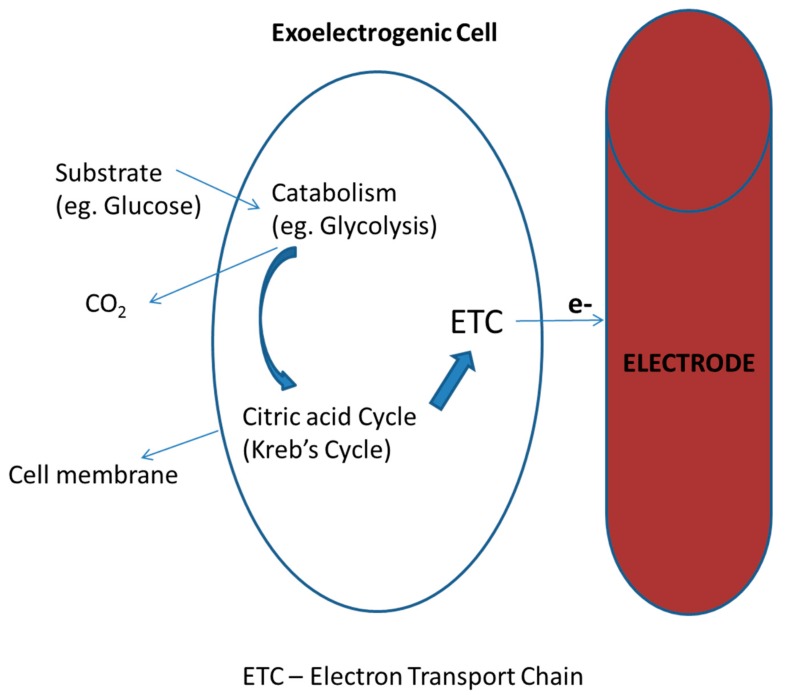
A general schematic diagram of the catabolic and respiratory pathway in exoelectrogens.

**Figure 3:**
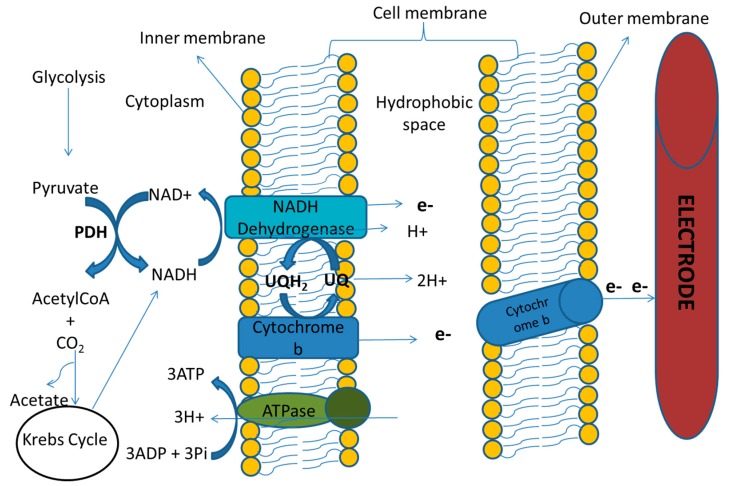
A Schematic diagram of a magnified view of the exoelectrogen’s cell membrane showing Extra-cellular Electron Transfer (EET) NADH-dependent catabolic pathway consisting of Pyruvate Dehydrogenase (PDH) and NADH. UQ is oxidized form of ubiquinone; UQH2 is reduced form of ubiquinone.

Cellular respiration is said to be complete when electrons are taken by a final electron acceptor in the Electron Transport Chain (ETC) to produce ATP [30]. This electron acceptor can be molecular oxygen (O_2_) as in aerobic respiration for oxidative phosphorylation or other soluble compounds, such as iron (especially Fe (III) oxides) and manganese (Mn (III/IV) oxides) compounds as in anaerobic respiration for substrate-level phosphorylation [17,30,32]. As seen in Figure 2, the exoelectrogenic cell takes up the organic substrate which then undergoes a catabolic reaction, such as glycolysis, to release some electrons and also an intermediate product like pyruvate. This pyruvate, after it has been broken down to acetyl CoA, enters into the Kreb’s cycle and the ETC to release more electrons with an electrode as the final electron acceptor. One of the major pathways of the ETC in the exoelectrogens is shown in Figure 3. One of the most important features of exoelectrogenic bacteria in the operation of MDC systems is the colonization of the anode electrode. The electrode serves as the final electron acceptor in the oxidation of the organic matter by the bacteria in the anodic chamber. This electronic transfer is exogenic, hence their name. Therefore, the energy gained by the bacteria will be the difference in the potential between the electron donor and the electrode. The electron acceptor present in the cathode chamber, which normally is either oxygen, ferricyanide (Fe (CN)^3^_6_), or protons (in the case of Microbial Electrochemical Cells), will become reduced by accepting the electrons that moved through the circuit. The maximum energy that an MDC can generate is calculated in an analogous way to the energy gained by microorganisms, based on the difference of the potentials between the electron donor and acceptor’s redox reactions.

### 2.2. Methods Electron Transports in METs

**Figure 4:**
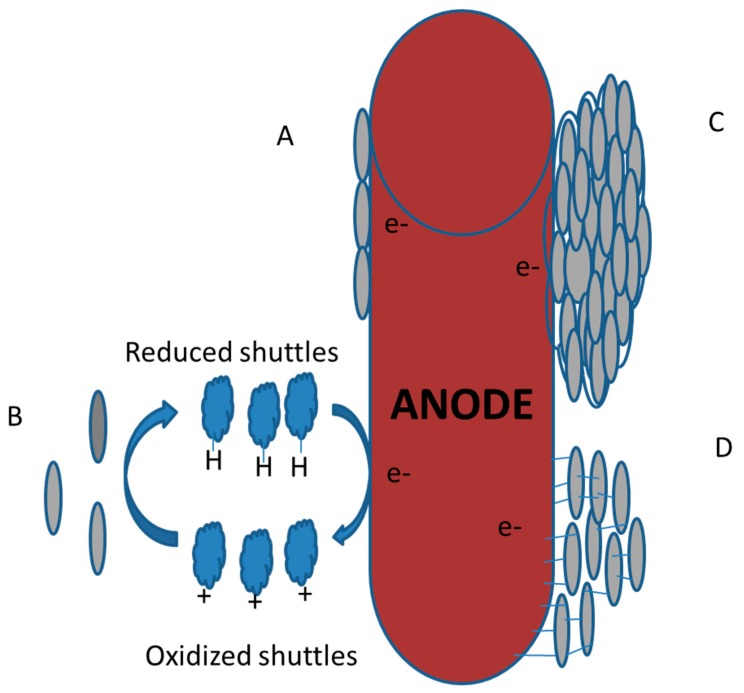
Main recognized mechanisms of electron transfer from exoelectrogens. (**A**) Direct electron transfer with c-type cytochromes (CTCs). (**B**) Soluble electron shuttles. (**C**) Electro-active biofilm formation. (**D**) Solid conductive matrix with nanowires or pili.

The exoelectrogenic bacteria transfer electrons to the anode in the anodic chamber in four major mechanisms as described in Figure 4:

(1) Direct contact of the exoelectrogens cells with the anode for electron transfer using C-Type Cytochromes (CTCs);
(2) The use of soluble electron shuttles such as flavins;
(3) By solid conductive components, such as nanowires or pili;
(4) Electro-active biofilm formation [27,32,38,39,40].

Direct contact of the exoelectrogenic bacteria with the anode is made possible by the proteins, such as Outer Membrane Cytochromes (OMCs), which receive the electrons in the surface of the cells. One family of these OMCs, called c-type cytochromes (CTCs), are heme-containing proteins that are located mostly in the outer cellular membrane and are part of the electron transport chain [20,30,40]. The high-potential electrode has been found in recent studies to stimulate the expression of respiratory genes in exoelectrogens, especially the genes for the outer membrane (OM) c-type cytochromes [41]. While the direct electron transfer through OMCs allows for few potentials loses between the outer membrane and the anode, the current density is severely limited due to the very amount of electrochemically active bacteria on the anode surface [39], allowing only the bacteria that surround the electrode (a monolayer biofilm) to be able to produce electric current.

The second mechanism that exoelectrogenic bacteria can employ is using molecules known as “electron shuttles”. They are small mediator compounds that receive the electrodes that are transported through respiration and are secreted to reach the anode and transfer the electrons. Chemically manufactured molecules can also be used as electron shuttles to enhance the facilitation of electron transfer to the anode [18,40]. According to Yang et al. (2012) [40], proper electron shuttles must be dissolvable, stable, reusable, environment-friendly, and have a proper potential. Electron shuttles that are generated inside the cells prove to be as effective and more sustainable in the operation of MFCs as compared to these manufactured mediators which are added exogenously to facilitate the transfer of electrons [18]. Examples of molecules endogenously produced to act as electron shuttles are flavins and phenazines [40]. While this method allows more bacteria to be able to transfer electrons, the produced current is limited because of the slow diffusion of reduced/oxidized electron shuttles [39], requiring high shuttle concentrations to overcome the mass transport limitations.

The third and most recently proposed method of electron transfer by exoelectrogens is the solid conductive matrix. The discovery of bacterial nanowires (electrically conductive pili [40]) and the capability of exopolymeric substances (EPSs) to act as semiconductors [39], allow exoelectrogens to form a thick biofilm to transport the electrons through the formed matrix, the rate of the transfer only being limited by its conductivity. Torres et al. (2010) [39] and Yang et al. (2012) [40] have recognized this method as the most efficient way to get high current densities and optimum bacterial growth, although the molecular composition of the nanowires and the matrix are yet to be extensively investigated and elaborated. A clear understanding of the conditions that favor this mechanism needs to be further studied.

The alignment of bacterial cells in a self-produced polymeric matrix on either biotic or abiotic surfaces is termed as a biofilm. The formation of electroactive biofilms has been extensively described and evaluated [32,42,43]. Within the biofilm, electrons are passed through adjacent cells until it reaches the electrode. A dense electro-active biofilm at the anode has been seen to produce optimum power in Bioelectrochemical systems (BES) [32]. Kumar et al., (2016) [32] also reported that the exoelectrogens in the mixed cultures can transport the electrons through direct interspecies electron transfer (DIET).

### 2.3. Pure Cultures and Mixed Cultures in MDC

It has been reported that the electricity generation capacity and the ability to adapt to the complex environment of BES with pure microbial cultures are worse than those of the systems with mixed microbial cultures [17,32,33]. However, pure cultures are very useful to elucidate the electron transfer mechanism at the microbiological and molecular levels and further reduce the complexity that comes from mixed cultures [30,33]. Production of electricity in the absence of exogenous mediators was first seen in *Shewanella putrefaciens* [36]. Shewanella spp. have different mechanisms to transfer electrons outside the cell. These mechanisms include the direct electron transfer by contact with the use of outer membrane cytochromes and also the transfer of electrons through conductive nanowires [44,45].

Most of the MDC systems use mixed cultures in their operations knowing the merits mixed cultures have over pure cultures in terms of species-diversity, metabolism, and performance as described in Table 1. Most mixed cultures usually exhibit predominant species that are easily adaptable to the environment of the tri-functional process in MDC systems. Though the predominant species produce the majority of the electrons through the oxidation of the organic matter in the anode chamber, other exoelectrogenic species produce electrons as well through their respiratory processes. This increases the capacity of power generation, enhancing wastewater treatment and desalination as compared to MDC systems with pure cultures which may use few mechanisms in electron transfer. Since these pure cultures use one or two of the electron transfer mechanisms, these mechanisms can be easily identified and the pure cultures can be genetically modified to maximize such mechanisms to increase electron production and oxidation of the organic matter in the system.

## 3. Performance Indicators of Exoelectrogens in MDCs

Exoelectrogens serve as primary determiners of the performance of the tri-functional process of the MDC systems. Their unique makeup requires certain conditions for growth and proper functioning. Several factors and parameters influence the growth, metabolism, and electron transfer mechanism of exoelectrogens, which in the end influence the general performance of the cell. Some of the major factors are discussed in detail in the subsections below. Besides, the structural integrity of the IEMs affected by exoelectrogens is highlighted. Table 2 summarizes the relationship between exoelectrogens and how varying conditions influence their performance.

### 3.1. Substrates and COD Removal

Most of the substrates used in MFC and MDC are in the form of butyrate, oxalate, glucose, and other easily degradable substrates [35,46,47,48]. Municipal, industrial, and domestic wastewater has also been mostly used as a substrate for the exoelectrogens in the anode chamber of MDC [12,13,14]. The mode of operation (batch, fed-batch semi-continuous, and continuous modes) at the anode chamber will depend on the configuration of the MDC system and the focus of the research [1,2,4,12,13,14,15]. Exoelectrogenic bacteria are substrate-specific, and the type of substrate used in an MDC system will determine the exoelectrogens needed to be used or to be predominant, its performance and the overall performance of the MDC system. As seen in Table 2, a 55% COD removal from domestic wastewater with a mixed culture having Proteobacteria being a predominant species at the anode chamber was reported by [12]. In another study conducted by them using Municipal wastewater, Actinobacteria was predominant at the anode chamber with 52% removal of COD. Shinde et al., (2018) [14] treated steel plant wastewater in a conventional MDC system with *Pseudomonas putida* in activated sludge at the anode chamber and recorded about 70% removal of COD. When pure cultures are used in MDC systems, their substrates should be well determined to greatly enhance their effective COD removal. A typical example is given in Table 2, in which the use of *Bacillus subtilis moh3* by Kalleary et al. (2014) in the anode chamber, containing 0.1% yeast extract with Malachite green dye, of a conventional MDC, resulted in a complete de-colorization. This was repeated with 0.1% yeast extract with Sunset yellow dye as the anolyte, which was also completely de-colorized by *Bacillus subtilis moh3* [15]. Thus, indicating the substrate specificity of the exoelectrogenic bacteria and their effective performance. There has been a wide range of substrates used in MDC, and it has been observed that certain exoelectrogens are predominant in mixed cultures in the presence of certain kinds of substrates. This has shown that the percentage of COD removal from the system is dependent on the type of substrate in the system and the predominant species needed to oxidize the substrate. The oxidation of these substrates, by the exoelectrogens, results in the release of electrons which are accepted by the anode, thereby causing the exoelectrogens to gain energy. The increase of the substrate level in the system may not necessarily increase the release of electrons since the exoelectrogens may have reached a saturation level, which may impede the release of electrons among other factors as well.

### 3.2. Electricity Output

The electrons released to the anode by the exoelectrogens pass through the circuit to the cathode to reduce O_2_ to H_2_O at the cathode chamber. The flow of the electrons in the circuit causes a power output that can be stored and measured. It has been observed that the rate of substrate oxidation by the exoelectrogens influences the power output. Hence, the higher the rate of substrate oxidation the higher the power output in an MDC system. Moreover, the ability of the anode to accept the electrons, from the exoelectrogens, is essential to the power output of the MDC system. Most anodes are now being designed to have a large surface area to accept electrons from the exoelectrogens thereby increasing the power output. Mixed cultures with predominant species produce more power output than single strains at the anode chamber of MDC systems. The inoculation of the anode chamber with a rich and diverse source of bacteria, such as wastewater or leachate, frequently produces the highest power outputs in MDCs. The power outputs that are produced by either pure or mixed cultures are mostly dependent on the specific construction, membrane and electrode spacing, and conductivity of the solution of the MDC rather than the specific bacterium or strain. Thus, power outputs produced by a pure or mixed culture in a cell cannot directly be compared with the power generation from another cell (pure or mixed culture) unless the MDC configuration, chemical composition, and other parameters are similar. Luo et al.’s (2012) record of 8.01 W/m^3^ from their study on Municipal wastewater [12] is the highest power output that gives details of the predominant species, *Actinobacteria*, in the mixed culture used in the anode chamber. It was higher than their other research with 3.6 W/m^3^ from domestic wastewater in a similar conventional MDC. It had *Proteobacteria* as predominant species in the mixed culture used in the anode chamber (as seen in Table 2). Both studies were operated in a fed-batch mode. In other BES, such as air-cathode MFC with a mixed culture in the anode, a high electrical output of 1.55 kW/m^3^ has been achieved which is higher than the use of pure culture [49].

The increase of electrical output also increased the rate of desalination in the experiments stipulated in Table 2. These electrical outputs were achieved by the release of electrons from the exoelectrogens through the oxidation of the organic matter in the anode chamber. This also enhanced COD removal in the systems. An electrical output as low as 488 mW/m^3^, produced by a pure culture, *Debaryomyces hansenii*, in a conventional MDC, was able to cause desalination of about 55.03% [13]. Thus, it shows an inter-relational dependence of the three basic processes in MDC greatly influenced by the exoelectrogens.

### 3.3. The Balance of pH

Apart from substrate composition, the pH level at the anode chamber also determines the predominant species in the mixed culture of exoelectrogens. Most exoelectrogens are seen to be effective and active at pH between 6.0 and 7.0 [10,12,13,14]. Certain strains such as *Bacillus subtilis moh3* have been seen to be effective at a pH of 4.0 [15]. A pH of 6.0–7.0 is the usual pH needed for the optimal performance and growth of exoelectrogens in MDC systems and their effective tri-functional process. Among other favorable conditions, it is useful to know the specific pH of an exoelectrogenic species to make it predominant in the mixed culture in the MDC system. The release and uptake of electrons and protons in the MDC chambers cause fluctuation of pH level, which affects the growth, and proper functioning of the exoelectrogens. Buffers have been used in stabilizing the pH levels in MDCs [12,13,14]. New MDC configuration known as recirculating Microbial Desalination Cell (rMDC) has also been made to tackle the issue of pH imbalances in the system [4,11,43,50].

### 3.4. Desalination

The electric potential gradient created by exoelectrogenic bacteria in BES desalinates water by driving ion transport through a series of ion-exchange membranes (IEMs). The third chamber, which is between the anode and cathode chambers, of the MDC system contains either seawater, saltwater (usually 35g/L) or brackish water [1,10,14,15,35,42]. In favorable conditions, the release of electrons by the exoelectrogens, through the circuit causes a potential difference, which enables the separation of NaCl, in the third chamber, into its constituent ions. The Na+ ions move into the cathode chamber through the Cathode Exchange Membrane (CEM) while the Cl^-^ ions move into the anode chamber through the Anode Exchange Membrane (AEM), as seen in Figure 1. This process is termed as desalination. Lefebvre et al. (2012) [51] reported that Cl concentrations, at the anode chamber, of up to 300 mM do not adversely affect power generation. However, the maximum power output is reduced by 12% at 500 mM Cl concentration and the increase of KCl concentrations in the system also increases power generations but only up to 300 mM [51]. This implies that at very high concentrations of anions produced exoelectrogenic microbial activity is affected while using a high content of organic substrates in the anolyte of anode chamber. Again, the type of exoelectrogen used also determines the effect of high salt concentrations. The activity of *Pelobacter propionicus* decreased and that of *Geobacter sulfurreducens* increased with increasing NaCl concentration in the anolyte, thus suggesting that a given salinity level may favor a particular type of exoelectrogen species only [52]. The use of mixed culture with predominant species at the anode chamber has been shown to produce effective desalination as compared to single strains of certain exoelectrogens [12,13,14,15]. In two of his research, Luo et al. (2012b) [10] recorded about 66% desalination in conventional MDCs with mixed cultures confirming the high efficiency of desalination in the system when mixed cultures are used.

### 3.5. Structural Integrity of Ion Exchange Membranes

Generally, there are two kinds of Ion Exchange Membrane (IEM) in MDCs: Cathode Exchange Membrane (CEM) (separates the desalination chamber from the cathode chamber and regulates the ionic exchange between these chambers) and Anode Exchange Membrane (AEM) (separates the desalination chamber from the anode chamber and regulates ionic exchange between these chambers). Formation of microbial biofilm occurs on the surface of the AEM facing the side of the anode chamber due to the presence of the microbial community, especially the exoelectrogens [12,17,31,40,52]. Biofilm formation on the surfaces of the AEM, which is termed as biofouling, normally occurs when the MDC system has been used continuously for a long duration [10]. The structural integrity of the IEM and its functional groups become compromised due to the growth of biofilm and deposition of organic matter in the anode chamber on its surface. This increases the internal resistance of the system and thus affects the efficiency of the tri-functional process of the MDC system [10,12]. This area of MDC has not been adequately researched and needs extensive investigation. Coating of the AEM, the use of nanomaterial in the make-up of the AEM among others might drastically reduce the biofilm formation on the surface of the AEM and enhance its structural stability and integrity which will extend its life and reuse, and increase efficiency in the MDC system.

## 4. Future Prospect

For any MDC system to be on its optimal performance, more research needs to be done on improving the efficiency of the exoelectrogens used in MDC. The use of genetic engineering will play a major role in enhancing certain strains that are predominant in mixed cultures. These strains can be engineered to be more efficient in COD removal, have a high affinity to the anode, and effectively reduce membrane fouling at the anode chamber, thereby increase desalination. It should be noted that these genetically modified strains will be more effective in mixed cultures than pure cultures. Further research needs to be done on enhancing the integrity of the IEMs used in MDCs to reduce the formation of biofilms on the surfaces of the IEMs. Moreover, MDC should be integrated with other processes, such as nanotechnology, wastewater treatment, and desalination systems, to make this technique economically feasible.

## 5. Conclusions

Exoelectrogens are some of the most important components of any MDC system and its performance determinants. Factors that influence their growth and survival, also directly or indirectly affect the performance of the MDC system. This review looked into the basic characteristics of exoelectrogenic bacteria, which are primary influencers in MDC, a comparative analysis of pure and mixed cultures used in the MDC, and factors that influence these exoelectrogens and the overall performance of MDC. The application of MDC for wastewater treatment at the industrial level could be an attractive alternative to reduce the cost of existing systems.

## Figures and Tables

**Table 1:** Differences in the performance of pure cultures and mixed cultures used in MDC Systems [17,30,40].

Pure Cultures	Mixed Cultures
Substrate specificity	Predominant species dependent on substrate
Uses one or few of the electron transfer mechanism	Combines several electron transfer mechanism
Very costly in isolating and preparation	Less costly in obtaining and preparation
Less efficient in electricity generation	More efficient in electricity generation
Known electron transfer mechanism which can be engineered for optimum performance	Unknown electron transfer mechanism being used.

**Table 2:** Taxa of exoelectrogens and performance in MDC Systems.

Exoelectrogens At Anode Chamber	Substrate	Mode of Operation	COD Removal at Anode Chamber	Desalination	pH of Anolyte	Temp. (°C) of Anolyte	Power Output	Configuration	References
*Debaryomyces hansenii*	Glucose	Batch	-	55.03%	6.5	-	488 mW/m^3^	Conventional MDC	[13]
Biofilm predominantly *Proteobacteria*	Domestic Waste Water	Fed-Batch	55%	<66%	-	-	3.6 W/m^3^	Conventional MDC	[10]
Biofilm predominantly *Actinobacteria*	Municipal Waste Water	Batch	52%	66%	6.0 ± 0.1	-	8.01 W/m^3^	Conventional MDC	[12]
*Pseudomonas putida* with activated sludge	Steel Plant Waste Water	Batch	70 ± 1.8%	-	7.0 ± 0.2	-	10.2 mW/m^2^	Multi-Chambered MDC	[14]
*Bacillus subtilis moh3*	0.1% yeast extract with Malachite green dye	Fed-Batch	Complete de-colorization	62.2 ± 0.4%	4.0–8.0	30.0	0.15 ± 0.05 W/m^3^	Conventional MDC	[15]
*Bacillus subtilis moh3*	0.1% yeast extract with Sunset yellow dye	Fed-Batch	Complete de-colorization	57.6 ± 0.2%	4.0–8.0	30.0	0.14 ± 0.03 W/m^3^	Conventional MDC	[15]
